# Critical appraisal of systematic reviews of intervention studies in periodontology using AMSTAR 2 and ROBIS tools

**DOI:** 10.4317/jced.60197

**Published:** 2023-08-01

**Authors:** Alexandre-Godinho Pereira, Carolina-Castro Martins, Julya-Ribeiro Campos, Sandro-Felipe-Santos Faria, Sarah-Queiroz Notaro, Tina Poklepović-Peričić, Lidiane-Cristina-Machado Costa, Fernando-Oliveira Costa, Luís-Otávio-Miranda Cota

**Affiliations:** 1 Department of Dental Clinics, Oral Pathology, and Oral Surgery, School of Dentistry, Federal University of Minas Gerais, Belo Horizonte, Minas Gerais, BrazilDepartment of Dental Clinics, Oral Pathology, and Oral Surgery, School of Dentistry, Federal University of Minas Gerais, Belo Horizonte, Minas Gerais, Brazil; 2 Department of Pediatric Dentistry, School of Dentistry, Federal University of Minas Gerais, Belo Horizonte, Minas Gerais, Brazil; 3 Department of Research in Biomedicine and Health, University of Split School of Medicine, Split, Croatia; 4 School of Dentistry, Newton Paiva University Center, Belo Horizonte, Minas Gerais, Brazil

## Abstract

**Background:**

Systematic reviews of intervention studies are used to support treatment recommendations. The aim of this study was to assess the methodological quality and risk of bias of systematic reviews of intervention studies in in the field of periodontology using AMSTAR 2 and ROBIS.

**Material and Methods:**

Systematic reviews of randomized and non-randomized clinical trials, published between 2019 and 2020, were searched at MedLine, Embase, Web of Science, Scopus, Cochrane Library, LILACS with no language restrictions between October 2019 to October 2020. Additionally, grey literature and hand search was performed. Paired independent reviewers screened studies, extracted data and assessed the methodological quality and risk of bias through the AMSTAR 2 and ROBIS tools.

**Results:**

One hundred twenty-seven reviews were included. According to AMSTAR 2, the methodological quality was mainly critically low (64.6%) and low (24.4%), followed by moderate (0.8%) and high (10.2%). According to ROBIS, 90.6% were at high risk of bias, followed by 7.1% low, and 2.4% unclear risk of bias. The risk of bias decreased with the increased in the impact factor of the journal.

**Conclusions:**

Current systematic reviews of intervention studies in periodontics were classified as low or critically low methodological quality and high risk of bias. Both tools led to similar conclusions. Better adherence to established reporting guidelines and stricter research practices when conducting systematic reviews are needed.

** Key words:**Bias, evidence-based dentistry, methods, periodontics, systematic review.

## Introduction

Systematic reviews (RSs) of intervention studies are considered of high level of scientific evidence, being used to raise evidence that can support treatment recommendations and public health strategies (1). As other study designs, SRs are subject to biases that can compromise their validity and quality of evidence (2). Some tools were developed to assess the methodological quality and risk of bias of SRs, such as AMSTAR 2 (A Measurement Tool to Assess Systematic Reviews 2) (3) an updated version of ASMTAR (Risk of Bias in Systematic Reviews) (4), the Cochrane Collaboration tool for risk of bias of SRs (5).

Some overviews in the periodontal field have assessed the methodological quality of SRs through AMSTAR, showing inconstant quality (6-9). One overview assessed the methodological quality of SRs using the AMSTAR 2 and the risk of bias through ROBIS, and demonstrated very low overall quality (10). Among 23 SRs, only 3 SRs on peri-implantitis therapy had high quality according to AMSTAR 2, and only one were judged as low risk of bias according to ROBIS (10). This low overall quality raised questions about the general quality of the available evidence from RSs in periodontology.

Hence, this overview aimed to: 1) describe the characteristics of SRs in periodontology; 2) assess if the certainty of the evidence is reported in these reviews; 3) assess the methodological quality using the AMSTAR 2; 4) assess the risk of bias using the ROBIS.

## Material and Methods

This methodological survey was designed and performed following the recommendations of the Cochrane Handbook for Systematic Reviews of Interventions (11) and was reported in accordance with the PRISMA checklist (12).

-Research question

What is the methodological quality and risk of bias of the SRs of intervention studies in periodontology published in 2019-2020?

-Eligibility criteria

Inclusion criteria were SRs of intervention studies – randomized (RCTs) and non-randomized clinical trials (nRCTs) – with or without meta-analysis, in the field of periodontology, indexed between October 1st, 2019 to October 1st, 2020. SRs that authors classified the studies as having prospective design were included as nRCTs. According to the Risk of Bias in Non-randomized Studies of Interventions (ROBINS-I), nRCTs are cohort studies in which intervention groups were allocated during the usual course of treatment instead of randomization (13). To be consistent, all non-randomized studies, nominated by authors as clinical trials, controlled clinical trials, prospective controlled trials, non-randomized prospective studies, prospective clinical studies, prospective controlled clinical studies and retrospective cohort studies, were classified as nRCTs.

Exclusion criteria were: (a) SRs not related to the field of periodontology, (b) narrative or scope reviews, clinical guidelines, editorials or expert opinion papers, SRs of case-control and cross-sectional studies with PECO question, case reports and case series, pilot, *in vitro* and/or animal studies.

-Search in databases

An expert in SRs (CCM) designed and verified the strategies searches, and one reviewer (AGP) searched the following databases: MedLine (Pubmed), Embase (Elsevier), Web of Science, Scopus, Cochrane Library and LILACS for articles indexed between October 1st, 2019 to October 1st, 2020, with no language restrictions. This time length is enough to represent the current status of the quality of evidence in periodontology in the previous years, as the average time between the last search for a SR and its publication varies between 8 (14) to 15 months (15), and the mean time between the protocol’s publication and the SR’s publication is about 16 months (16).

Grey literature was searched in OpenGrey, GreyLit and Google Scholar. A hand search was performed in the references list of selected articles, and in the main journals of periodontology found in the Journal Citation Reports (JCR) in the category “Dentistry, Oral Surgery and Medicine”: Journal of Clinical Periodontology, Journal of Periodontology, Journal of Periodontal Research, International Journal of Periodontics & Restorative Dentistry, Journal of Periodontal and Implant Science, Periodontology 2000.

Additional information of search strategies, including search terms, is detailed in the supplementary material (Supplement 1) (http://www.medicinaoral.com/medoralfree01/aop/jced_60197_s01.pdf).

-Studies selection

Two pairs of independent reviewers screened studies based on titles and abstracts and then full text (AGP and SFF; JRC and LCMC). The reviewers were trained with a set of 10% of studies in each phase. In cases of less than 80% of agreement, additional rounds of training were carried out until reaching the necessary standard for each step. After reviewers achieved at least 80% of agreement, they underwent the screening process with the remaining of studies. The Rayyan platform (17) was used for studies screening. In cases of disagreement, an expert reviewer was consulted (CCM).

-Data extraction and assessment of methodological quality

Data extraction, assessment of methodological quality and risk of bias were performed through the AMSTAR 2 (3) and ROBIS (5) tools by four pairs of independent reviewers (AGP and SFF; AGP and LCMC; JRC and SQN; CCM and TPP), using Excel spreadsheet editor. Reviewers were trained by two reviewers (AGP and CCM), the second one with broad experience in systematic reviews methodology. Again, the reviewers underwent as many rounds of training as necessary, until reaching 80% of agreement. All disagreements were solved by discussion and consensus. If consensus was not achieved, the principal investigator made the final decision.

General data were extracted from the articles, and the list of the extracted data is available in the supplementary material (Supplement 2) (http://www.medicinaoral.com/medoralfree01/aop/jced_60197_s02.pdf). We uploaded the SRs protocols from the registration platform to compare with the published review, and extracted the JCR impact factor and the h-5 index of the journals from the JCR and Google Scholar Metrics, respectively.

Disagreements during this step were resolved between the pair of reviewers. If disagreement persisted, the principal investigator was responsible for reaching a final consensus. Two reviews in Mandarin were translated using a translation tool.

-Statistical analysis

Data was entered in IBM SPSS Statistics for Windows version 25 (Armonk, NY: IBM Corp.) for descriptive analyses. We calculated the relative and absolute frequencies for categorical variables, and mean, standard deviation and minimum/maximum values were provided for continuous variables. Analyses were performed considering all SRs and stratified by: SRs with RCTs and nRCTs, SRs with RCTs only and the impact factor of the journals (<3, ≥3 <6, ≥6).

## Results

-Literature search

**Figure 1:**
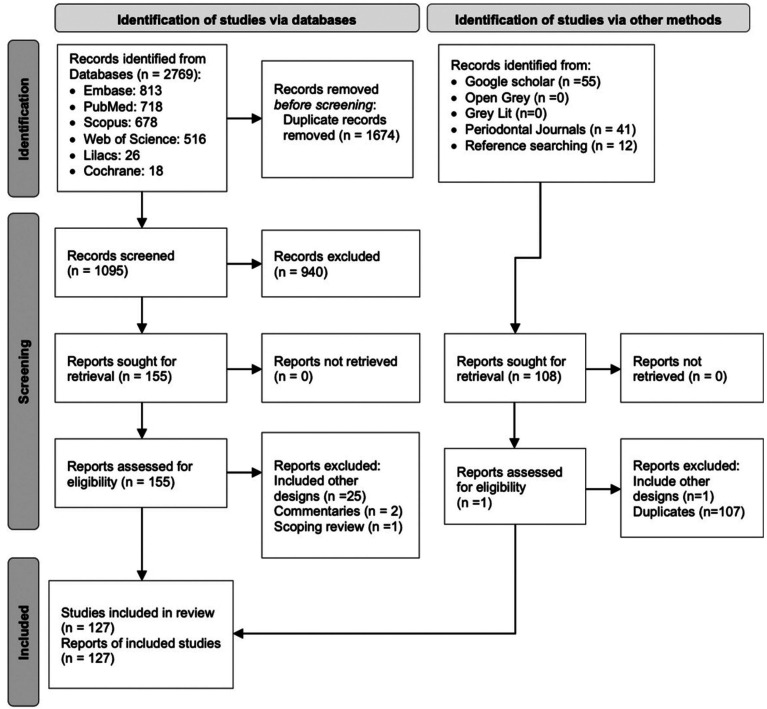
PRISMA flowchart.

One hundred twenty-seven SRs were included. Figure 1 shows the screening process. A list of excluded studies with reasons for exclusion is available on (Supplement 3) (http://www.medicinaoral.com/medoralfree01/aop/jced_60197_s03.pdf) and a list of references of the included RSs is available in (Supplement 4) (http://www.medicinaoral.com/medoralfree01/aop/jced_60197_s04.pdf).

-Characteristics of the studies

Studies characteristics are presented in Tables S1-S4 (Supplement 5) (http://www.medicinaoral.com/medoralfree01/aop/jced_60197_s05.pdf). The main language of publication was English (n= 124; 97.6%). The most common journals where the SRs were published were: Journal of Clinical Periodontology (n=17; 13.4%), Clinical Oral Investigations (n=10; 7.9%) and BMC Oral Health (n=9; 7.1%). Only 34.6% (n=44) of SRs were open access, and 66.1% (n=84) were available without restriction. Ninety-three (73.2%) journals had JCR impact factor with mean of 4.4±2.5. One-hundred three (96.8%) journals had h5 index, with an average of 46.1±29.3 (1.6 – 9.3).

-Origin and authorship

The number of authors ranged from 1 to 24 (mean 5.3 ± 2.6). Twenty-seven per cent of SRs had authors from different continents sharing the authorship (n=34). Few SRs included an epidemiologist (n=6, 4.7%), a librarian (n=9, 7.1%) and a biostatistician (n=9, 7.1%) in the research team.

-Main topics of study

The main intervention topics addressed by SRs were related to: antiseptics (n=23; 18.1%), soft tissue regeneration (n=20; 15.7%), periodontal photomedicine (n=18; 14.2%), antimicrobials (n=14; 10.9%) and guided bone regeneration (n=13; 10.2%).

The main periodontal conditions treated were: periodontitis (n=37; 29.1%), gingivitis and plaque/oral hygiene (n=33; 26.0%), gingival recessions (n=14; 11.0%), peri-implant diseases (n=13; 10.2%) and periodontal bone defects (n=12; 9.4%).

-Funding and conflict of interest

The main source of funding was: own financing (n=48; 37.8%), government/university (n=45; 35.4%) and industry (n=4; 3.1%). Twenty-seven RSs (21.3%) did not report funding sources. One-hundred eight SRs (85.0%) reported not having conflict of interest.

-Protocol, register and PRISMA

More than a third of the SRs (n=48; 37.8%) did not mention a study protocol. Among the 79 SRs that reported a protocol, 71 (89.9%) and 8 (10.1%) had registered and non-registered protocols, respectively. The most common registration platform was the International Prospective Register of Systematic Reviews (PROSPERO) (n=61; 85.9%).

-Searches and eligibility restrictions

A mean of 3.6 (±1.5) databases were searched, and 1.2 (±1.5) databases for grey literature. A manual search on the articles’ references was performed by 70.9% (n=90) of SRs, and 41.7% (n=53) of SRs manually searched in journals’ interest area. The most common databases searched were: MedLine (n=125; 98.4%), Cochrane Library (n=97; 76.4%), Embase (n=70; 55.1%), Web of Science (n=40; 31.5%), Scopus (n=38; 29.9%), Lilacs (n=16; 12.6%) and CINAHL (n=10; 7.9%).

-Number of studies and the presence of meta-analysis

The mean number of studies included per SR was 15.9±15.7 (range of 2–91), being 15.6±15.7 (1–91) RCTs and 0.4±1.3 (0–12) nRCTs. The total number of participants ranged from 67 to 13,426 individuals and the mean number of analysed outcomes was 4.6±2.9 (1–19). Most SRs (n=96; 75.6%) presented meta-analysis, 13 (10.2%) presented network meta-analysis, and 8 (6.3%) had a meta-regression. On average, 14.3±16.3 (2–105) studies were included in the meta-analyses and 3.2±1.9 (1–11) outcomes were meta-analysed SR.

Assessment of risk of bias and certainty of the evidence

The main risk of bias tool used for RCTs was the Cochrane tool (n=103; 81.1%) and ROBINS-I for nRCTs (n=17; 23.5%). Thirty-two (25.2%) SRs assessed the certainty of the evidence using the GRADE approach, and among them, 12 (37.5%) assessed the certainty of the evidence following the GRADE approach guide, 7 (21.9%) partially followed it, and 13 (40.6%) deviated from the GRADE approach guide.

-AMSTAR 2

The overall AMSTAR 2 methodological quality of all SRs was classified as critically low (n=82, 64.6%), low (n=31, 24.4%), moderate (n=1, 0.8%) and high (n=13, 10.2%) (Table 1,  Table 1 cont.).

**Table 1:**
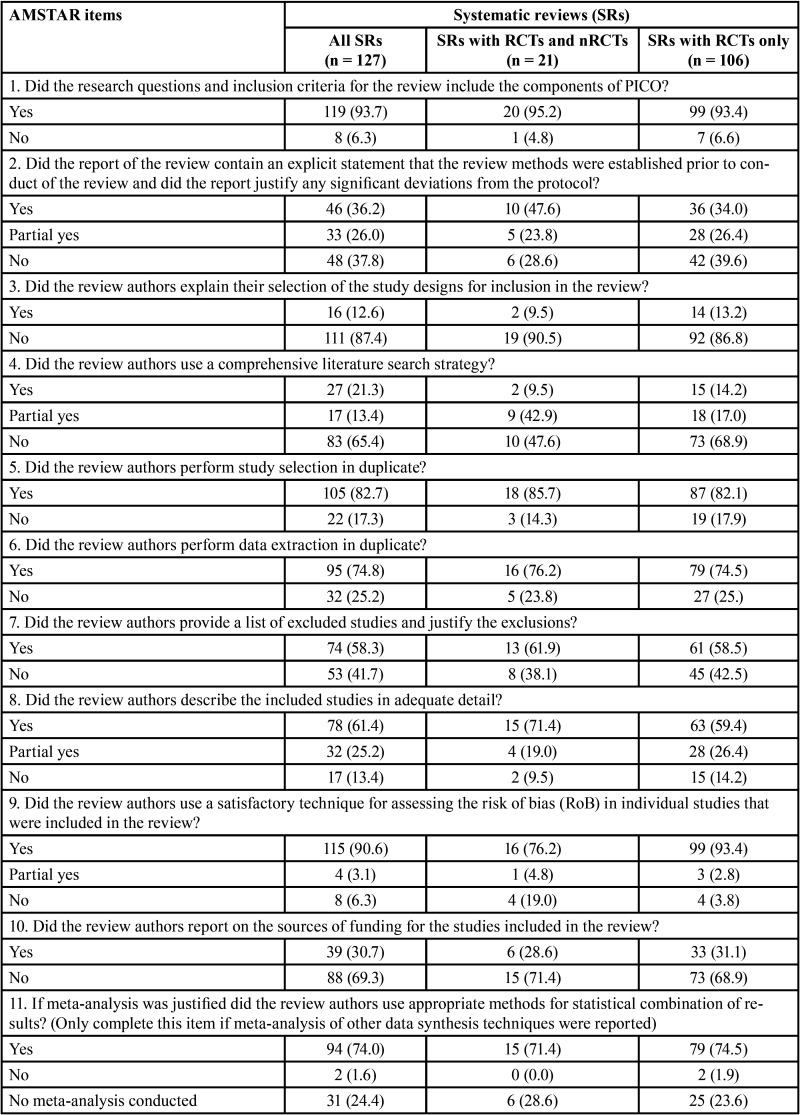
Methodological quality assessment through AMSTAR 2 according to the type of studies included in the systematic reviews.

**Table 1 cont.**
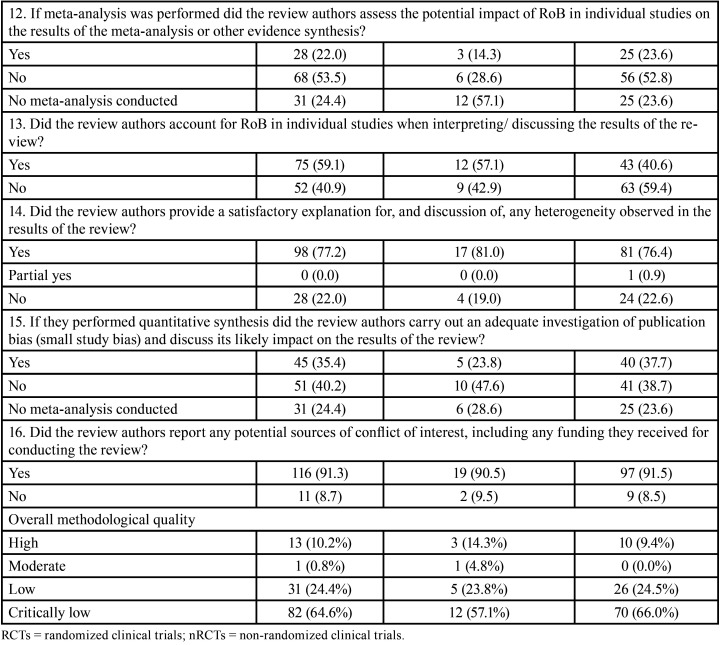
Methodological quality assessment through AMSTAR 2 according to the type of studies included in the systematic reviews.

The items 1 (components of PICO), 5 and 6 (study selection and data extraction in duplicate), 9 (satisfactory assessment of risk of bias), 11 (appropriate methods for meta-analysis), 14 (satisfactory discussion of heterogeneity) and 16 (report of sources of conflict of interest) received positive answers in more than 70% of SRs. The items with the highest percentage of overall negative responses were: 3 (reason for selection of certain study designs; 87.4%), 10 (report of funding sources for the included studies; 67.7%) and 4 (careful search of the literature; 65.4%). Five items considered critical according to AMSTAR 2 had large percentage of negative assessments: 2 (presence of protocol and justification for its modifications; 37.8%), 4 (careful literature search; 65.4%), 7 (list of excluded articles with justifications; 41.7%), 13 (consideration of the risk of bias in individual studies; 40.9%) and 15 (investigation and discussion of the impact of publication bias; 40.2%).

It is important to note that when analyses were performed considering the impact factor of the journal, the overall methodological quality was classified as high in ~30% of SRs in journals with impact factor ≥6. A high percentage of positive answers were also observed in the higher impact factor journals (Table 2- Table 2 cont.-1). No expressive differences were observed when evaluating SRs according to the design of the included studies (Table 1,  Table 1 cont.).

**Table 2:**
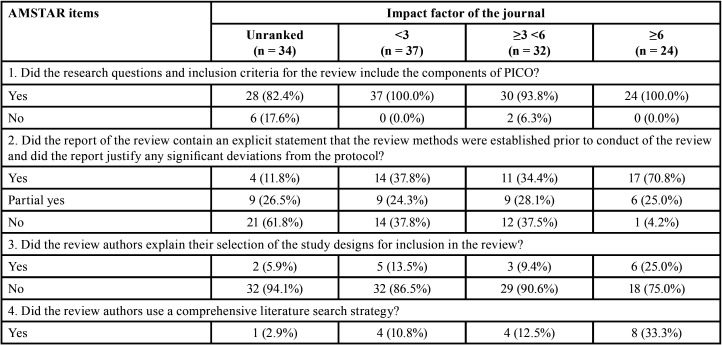
Methodological quality assessment through AMSTAR 2 according to the impact factor of the journals.

**Table 2 cont.**
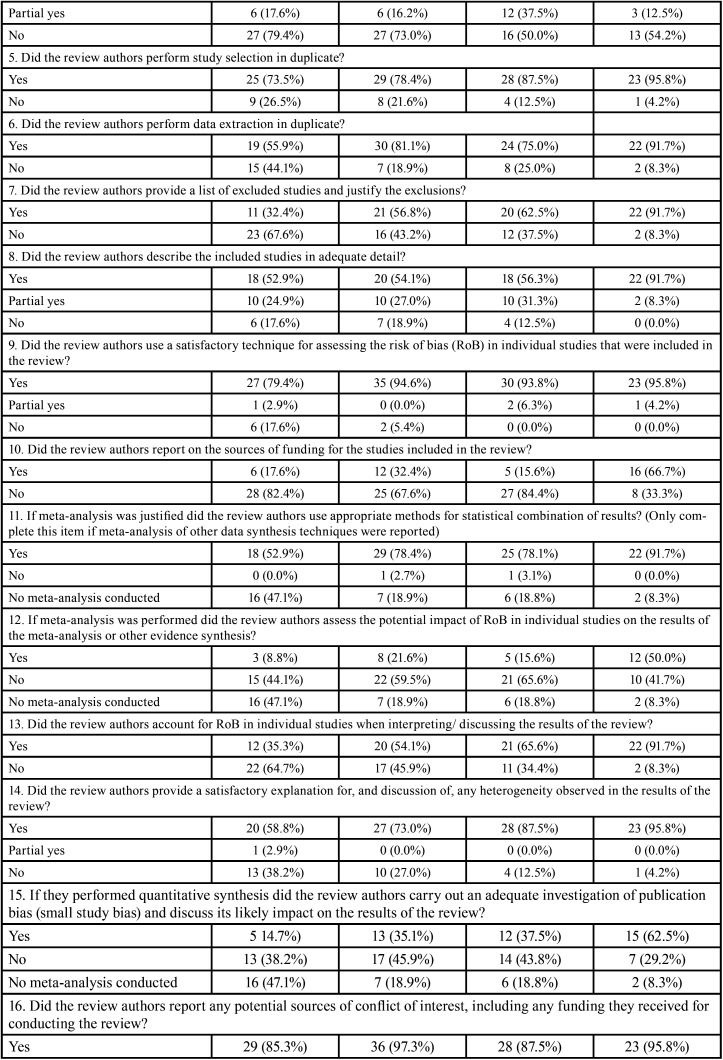
Methodological quality assessment through AMSTAR 2 according to the impact factor of the journals.

**Table 2 cont.**
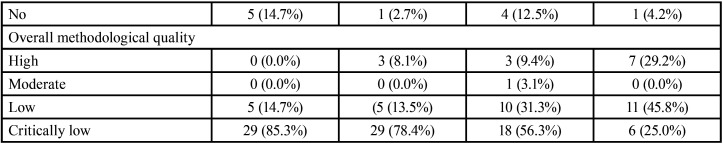
Methodological quality assessment through AMSTAR 2 according to the impact factor of the journals.

-ROBIS

The overall ROBIS evaluations considered 113 (90.6%) SRs to be at high risk of bias, 11 (7.1%) at low risk and 3 (2.4%) at unclear risk of bias (Table 3- Table 3 cont.-2).

**Table 3:**
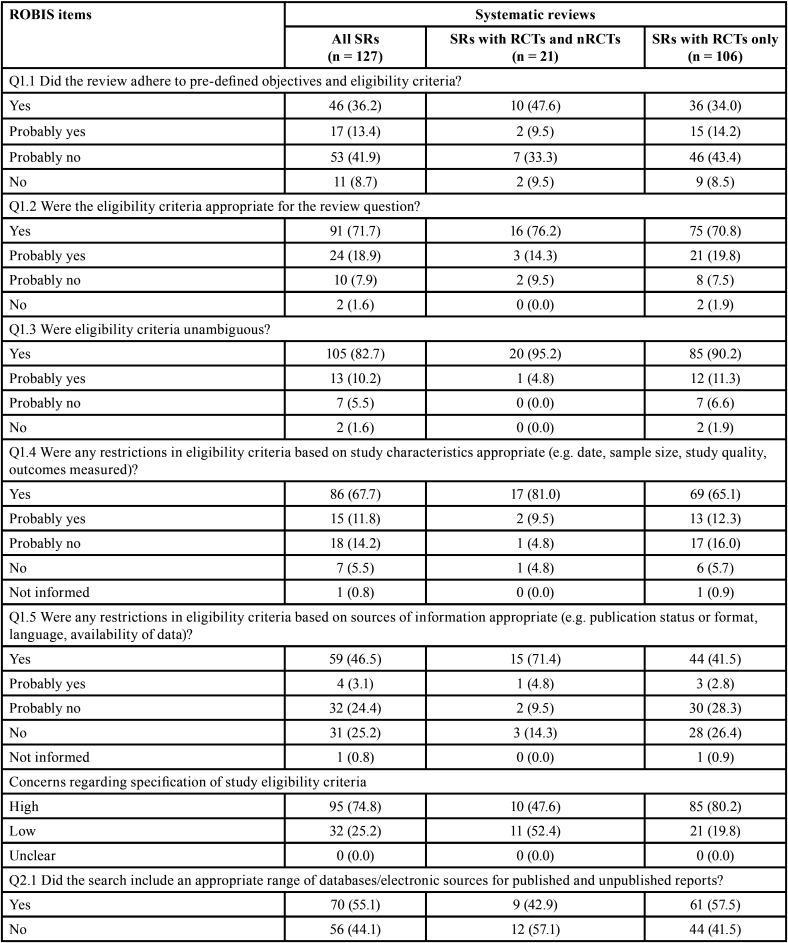
Risk of bias assessment through ROBIS according to the type of studies included in the systematic reviews.

**Table 3 cont.**
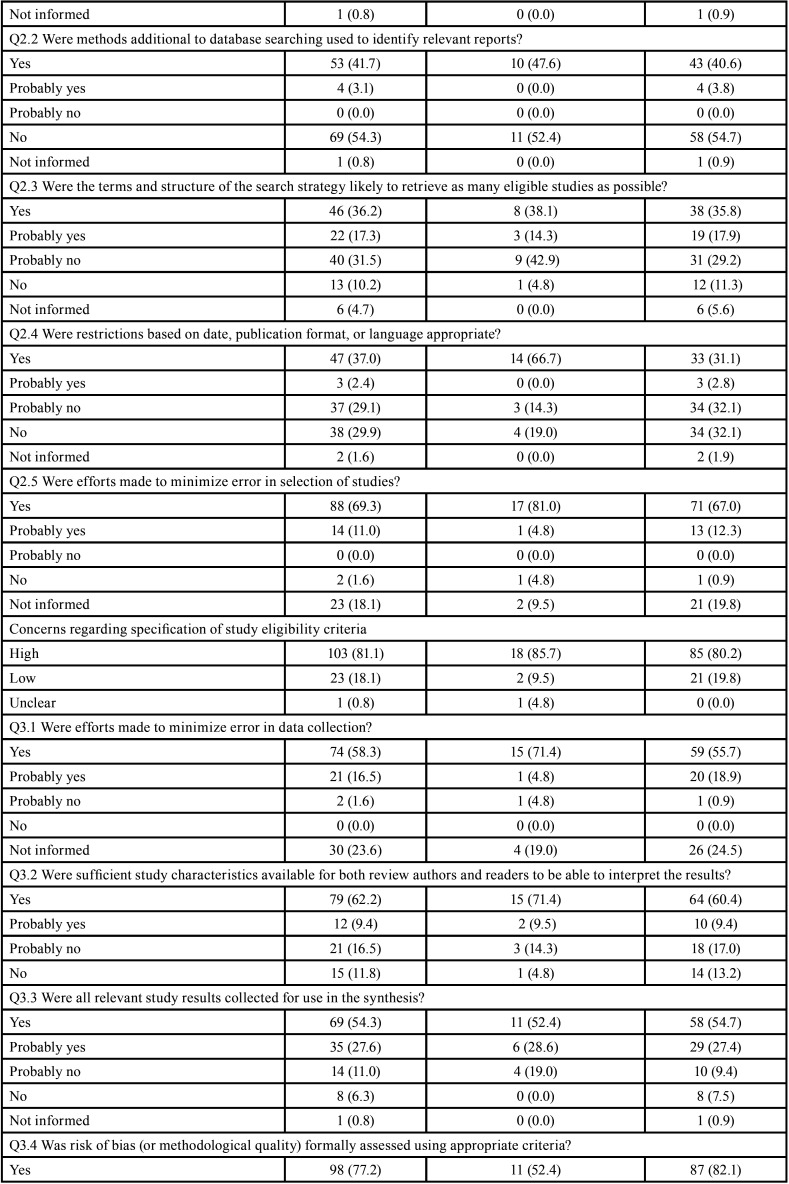
Risk of bias assessment through ROBIS according to the type of studies included in the systematic reviews.

**Table 3 cont.-1:**
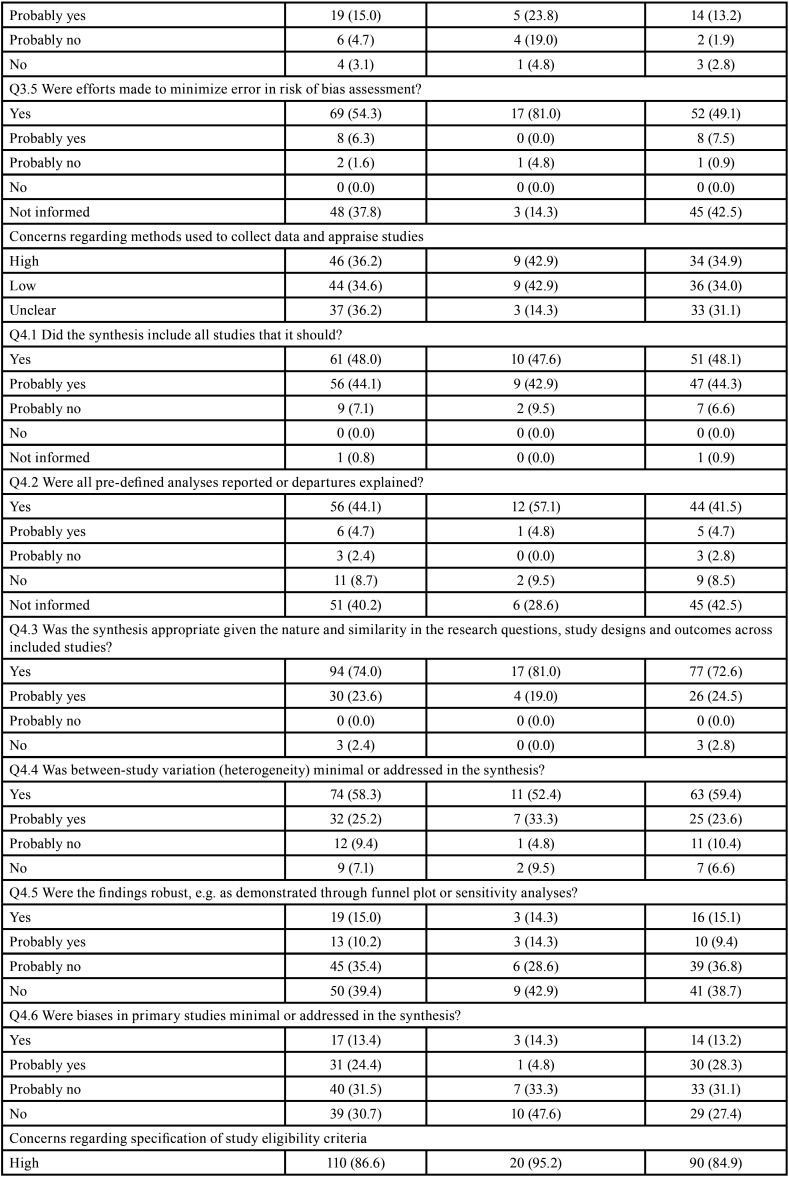
Risk of bias assessment through ROBIS according to the type of studies included in the systematic reviews.

**Table 3 cont.-2:**
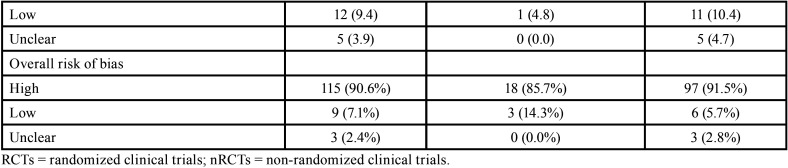
Risk of bias assessment through ROBIS according to the type of studies included in the systematic reviews.

Seventy-five per cent (n=95) SRs were judged with high risk on domain 1 (study eligibility criteria), and 25.2% (n=32) were judged as low risk of bias. The main issues were unjustified absence or deviation from the protocol (Q1.1; n=64; 50.6%) and unjustifiable restrictions in the eligibility criteria (Q1.5; n=63; 49.6%).

In domain 2 (identification and selection of studies), 81.1% of SRs (n=103) were at high risk and 18.1% (n=23) low risk of bias. The main issues were related to deficiencies in the literature searching in the main databases (Q2.1; n=56; 44.1%) and in the complementary searches (Q2.2; n=69; 54.2%).

The domain 3 (data collection and study appraisal), 36.2% of RSs (n=46) were at high risk of bias, 34.6% (n=44) low risk of bias and 29.1% (n=37) were at unclear risk of bias. The main issues were related to the lack of details of the included studies (Q3.2; n=36; 28.3%) and lack of the inclusion of relevant study results (Q3.3; n=22; 17.3 %). It is noteworthy that this was the domain with the highest percentage of undefined risk of bias, mainly due to use of an inappropriate risk of bias tool (Q3.4; n=48; 37.8%) and lack of independent reviewers to extract data (Q3.1; n=30; 23.6%).

The domain 4 (synthesis and findings) accounted with the highest overall risk of bias among the 4 domains: 110 (86.6%) SRs had high risk, while 12 (9.4%) SRs had low risk and 5 (3.9%) had unclear risk of bias. The main issues were the lack additional analysis or synthesis to test robustness of the results (Q4.5; n=95; 74.8%) and lack of assessment of the high risk of bias studies in the synthesis of results (Q4.6; n=79; 62.2%).

No expressive differences were observed when SRs were evaluated according to the design of the included studies (Tables 3). However, the risk of bias decreased with the increase of the impact factor of the journal ( Table 4). Detailed ROBIS assessments such as concerns regarding study eligibility criteria, methods used to collect data and appraise studies and the synthesis and findings also decreased with the increase of the impact factor of the journal ( Table 4- Table 4 cont.-2).

**Table 4:**
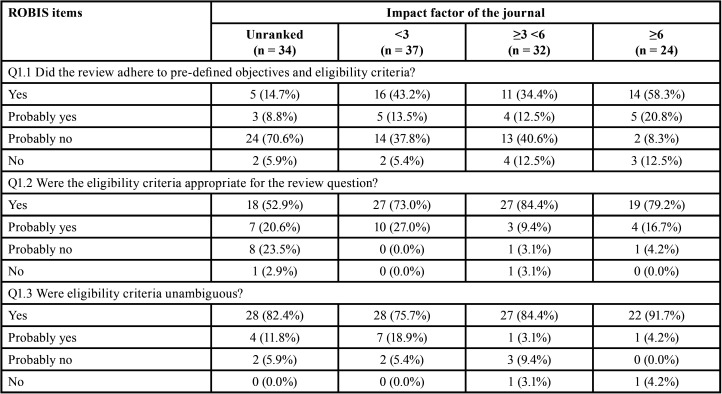
Risk of bias assessment through ROBIS according to the impact factor of the journals.

**Table 4 cont.**
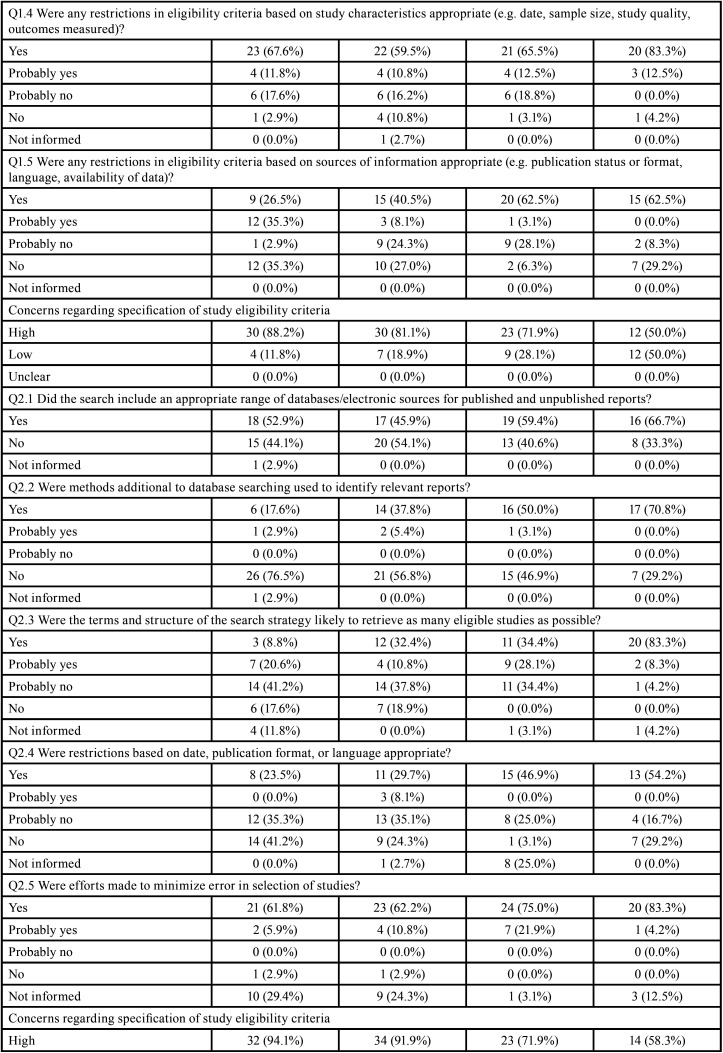
Risk of bias assessment through ROBIS according to the impact factor of the journals.

**Table 4 cont.-1:**
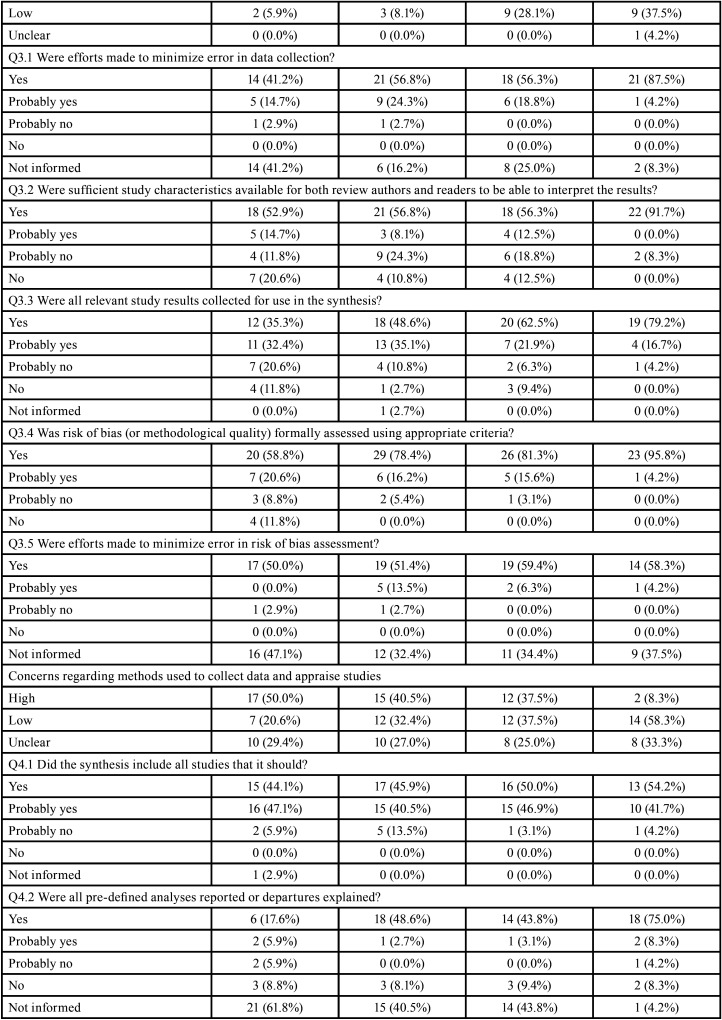
Risk of bias assessment through ROBIS according to the impact factor of the journals.

**Table 4 cont.-2:**
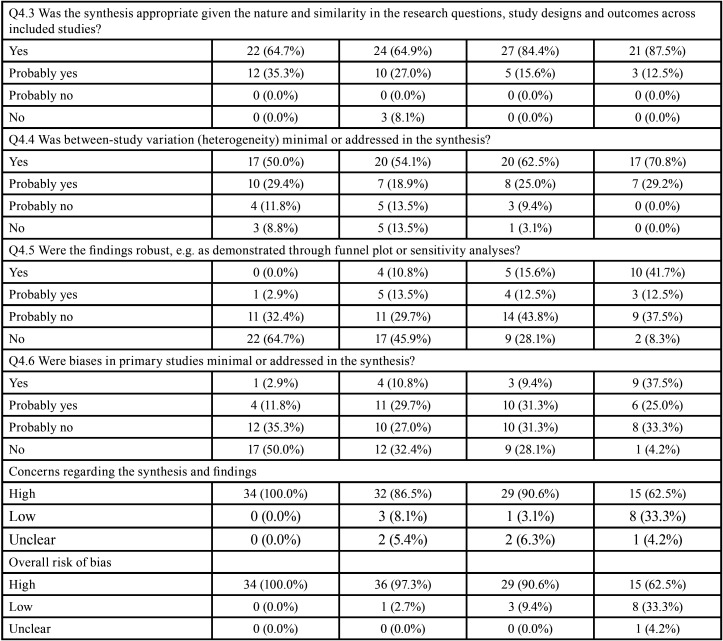
Risk of bias assessment through ROBIS according to the impact factor of the journals.

## Discussion

The majority of SRs were classified as high risk of bias according to the ROBIS that agreed with the low methodological quality of the AMSTAR 2. It seems that both tools can indicate similar results as they point out in the same direction. This is in accordance with a recent review classifying SRs in dentistry as low and critically low quality (18)

A wide variety of methodological deficiencies resulted in the classification of SRs as having high risk of bias. The absence or unjustified changes of the study protocol was the most important issue according to both tools. The prior creation and registration of a protocol is essential for ensuring the transparency of study methods and allowing adequate peer review of the proposed methodology, thus avoiding the selective reporting bias (11).

The deficiency of search strategies was another important identified bias. Search strategies for SRs should be as extensive as possible, without unjustifiable restrictions, including searches in the references of selected studies and in clinical trial registries. Additionally, complementary searches constitute an important source for the identification of potential studies. Its absence or unjustified restrictions increases the possibility of publication, language and selection biases, among others (11).

Among the nine SRs that included a librarian on the research team, 77.8% had high methodological quality searches when assessed by the AMSTAR 2, in contrast to 17.8% of high-quality searches in SRs not including librarians. The inclusion of librarians, although not mandatory, is beneficial as it provides guidance at various stages of the research, such as in the processes of designing search strategies and is associated with more reproducible searches and improved methodological reporting in dental medicine SRs (19).

The processes of selection, data extraction and assessment of the risk of bias, which should be ideally carried out independently by more than one reviewer, were presented incompletely in most of the SRs. Cross-checking or duplicate selection processes, data extraction and assessment of risk of bias can reduce biases, as well as the potential subjectivity of one single reviewer (20).

In addition to factors associated with methodological processes, the lack of robustness of the results and excessive bias in primary studies also lead to negative classification through the ROBIS assessment. Findings from SRs, especially those with meta-analyses, must be evaluated through complementary tests to assess its robustness, such as sensitivity tests, subgroup analysis, meta-regression and funnel plots (5). Few studies have proven the robustness of their findings, and the absence of such tests can result in false positive inferences in a meta-analysed result, leading the reader to believe in ineffective treatments.

It was reported that 68% of RCTs in the field of dentistry had an unclear or high risk of bias, according to the Cochrane risk of bias domains (21). If SRs do not test the robustness by meta-analytic approaches such as sensitivity analysis and meta-regression, the overall evidence may be biased. The inclusion of non-randomized intervention studies in the SRs might be considered an indication of acceptance of less-than-adequate research designs for intervention studies leading to low methodological quality or high risk of bias classifications. Nevertheless, no expressive differences were observed when SRs were evaluated according to the design of the included studies.

The vast majority of SRs were of low and critically low quality when assessed by the AMSTAR 2 and judged as high risk of bias by the ROBIS. Overall, these two instruments led to similar conclusions in 93.7% of the assessments, although they are intended for different purposes. The first one is designed to assess the methodological quality of RS, or if the important aspects of the methods are being full filled (3). The second one can detect the risk of bias, so, although the SRs had full filled one item, it does not mean that is free of bias (5). This high agreement is probably due to the overlapping questions between these instruments (22), as well as the low general methodological quality of the SRs analysed.

The main source of SRs was the collaboration among authors from different continents (26.8%) and most SRs (97.6%) were published in English. This trend demonstrates the globalization of world science with authors from different countries resulting in international partnerships, exchange of knowledge and resources between research groups and a greater visibility of scientific research (23).

Regarding the scope of the journals, 44.9% of SRs were published in general dental journals. This can be partially explained by the high percentage of studies (26%) whose interventions aimed at improving oral hygiene habits (plaque reduction and gingivitis), areas of common interest in most dental specialties. In addition, it is important to note that some periodontology journals are no longer accepting submissions of reviews. It was recently reported that there are no significant differences between moderate/high and low/critically low methodological quality SRs in dentistry regarding publication year, continent, dental specialty and the impact factor of the journal (18). On the contrary, the increase in the impact factor of the journal decreased the risk of bias according to ROBIS in the present study.

Few RSs (7.9%) did not mention conflict of interest in the paper at all or did not mention about funding (21.3%). The presence of financial ties can be associated with positive outcomes in RCTs (24). In addition, a survey of 3,247 scientists funded by the US National Institutes of Health showed that 15.5% admitted to altering a study’s design, methods, or results in response to pressure from funding sources (25). Thus, reporting potential conflicts of interest and funding sources is mandatory in scientific publications, as they aim to demonstrate the transparency and impartiality of the researchers who carry out the studies (11).

Only a quarter of SRs assessed the certainty of evidence using the GRADE approach. The assessment of the certainty of the evidence is important to help interpreting the results. As it is a more conservative approach, it can help to avoid misleading conclusions (26). Therefore, any SRs of intervention, independent of the field of science, should add the analysis of the certainty of the body of evidence in their methods (26).

Methodological and structural variability among systematic reviews have been observed and the quality of some studies is expected to vary (7). Notwithstanding the systematic and stringent approaches, not all systematic reviews are conducted and reported in the same manner and high methodological quality are uncommon according to specific checklists (7-10). Quality assessments of systematic reviews are quite recent and researches should consider some guidelines when designing, conducting and reporting their reviews.

In the contemporary scientific scenario, it has been speculated that some issues may influence the quality, reliability and bias of currently scientific research such as the pressure for scientific publication, large volume of articles, predatory journals, quality of the peer review process, among others (27-33). It was also reported that the dental literature has been increasingly reviewed on various topics leading to SRs with questionable clinical or scientific value in terms of up-to-date information to advance knowledge (34). Overall, researches should critically reflect on these issues in order to their scientific production be aligned to core principles of evidence-based dentistry. Guidelines and quality assessment tools may be helpful to identify topics to be improved.

Some limitations of the present study should be discussed. It had three pairs of independent reviewers, which may have resulted in different classifications by the peers. However, in order to establish solid classification criteria and to achieve high levels of agreement, four training and calibration sessions were conducted using the guidance documents of AMSTAR 2 (3) and ROBIS (5). A certain degree of variability in inter-examiner agreement was previously demonstrated (22,35). This methodological review is strong as it is the first one that raised the methodological quality using the new AMSTAR 2 together with ROBIS for risk of bias. Moreover, we extracted data of several characteristics of included SRs that are detailed in the supplementary material.

## Conclusions

Most SRs of intervention studies in periodontology were classified as low methodological quality and high risk of bias. Methodological quality increased and risk of bias decreased with the increase in the impact factor of the journals. Although designed for different purposes, both AMSTAR 2 and ROBIS could lead to similar directions. Efforts should be direct to better adherence to reporting guidelines and stricter research practices when conducting SRs. AMSTAR 2 and ROBINS could help the authors to plan the protocol and the reporting of their SRs.

## References

[1] Cuello-Garcia CA, Morgan RL, Brozek J, Santesso N, Verbeek J, Thayer K (2018). A scoping review and survey provide the rationale, perceptions, and preferences for the integration of randomized and nonrandomized studies in evidence syntheses and GRADE assessments. J Clin Epidemiol.

[2] Glenny AM, Esposito M, Coulthard P, Worthington HV (2003). The assessment of systematic reviews in dentistry. Eur J Oral Sci.

[3] Shea BJ, Reeves BC, Wells G, Thuku M, Hamel C, Moran J (2017). AMSTAR 2: a critical appraisal tool for systematic reviews that include randomised or non-randomised studies of healthcare interventions, or both.. BMJ (Clinical research ed).

[4] Shea BJ, Grimshaw JM, Wells GA, Boers M, Andersson N, Hamel C (2007). Development of AMSTAR: a measurement tool to assess the methodological quality of systematic reviews. BMC Med Res Methodol.

[5] Whiting P, Savović J, Higgins JP, Caldwell DM, Reeves BC, Shea B (2016). ROBIS: A new tool to assess risk of bias in systematic reviews was developed. J Clin Epidemiol.

[6] Faggion CM Jr, Giannakopoulos NN (2013). Critical appraisal of systematic reviews on the effect of a history of periodontitis on dental implant loss. J Clin Periodontol.

[7] Elangovan S, Avila-Ortiz G, Johnson GK, Karimbux N, Allareddy V (2013). Quality assessment of systematic reviews on periodontal regeneration in humans. J Periodontol.

[8] Hasuike A, Iguchi S, Suzuki D, Kawano E, Sato S (2017). Systematic review and assessment of systematic reviews examining the effect of periodontal treatment on glycemic control in patients with diabetes. Med Oral Patol Oral Cir Bucal.

[9] Natto ZS, Hameedaldain A (2019). Methodological Quality Assessment of Meta-analyses and Systematic Reviews of the Relationship Between Periodontal and Systemic Diseases. J Evid Based Dent Pract.

[10] Hasuike A, Ueno D, Nagashima H, Kubota T, Tsukune N, Watanabe N (2019). Methodological quality and risk-of-bias assessments in systematic reviews of treatments for peri-implantitis. J Periodontal Res.

[11] Higgins JPT, Thomas J, Chandler J, Cumpston M, Li T, Page MJ (2021). Cochrane Handbook for Systematic Reviews of Interventions version 6.2. In vitro comparison between Fluorinex® method and traditional topical fluoridation.

[12] Page MJ, McKenzie JE, Bossuyt PM, Boutron I, Hoffmann TC, Mulrow CD (2021). The PRISMA 2020 statement: an updated guideline for reporting systematic reviews. BMJ (Clinical research ed).

[13] Sterne JA, Hernán MA, Reeves BC, Savović J, Berkman ND, Viswanathan M (2016). ROBINS-I: a tool for assessing risk of bias in non-randomised studies of interventions. BMJ (Clinical research ed. ).

[14] Beller EM, Chen JK, Wang UL, Glasziou PP (2013). Are systematic reviews up-to-date at the time of publication?. Systematic Reviews.

[15] Sampson M, Shojania KG, Garritty C, Horsley T, Ocampo M, Moher D (2008). Systematic reviews can be produced and published faster. J Clin Epidemiol.

[16] Andersen MZ, Fonnes S, Andresen K, Rosenberg J (2021). Most published meta-analyses were made available within two years of protocol registration. Eur J Integr Med.

[17] Ouzzani M, Hammady H, Fedorowicz Z, Elmagarmid A (2016). Rayyan-a web and mobile app for systematic reviews. Syst Rev.

[18] Pauletto P, Polmann H, Réus JC,  Oliveira  JMD,  Chaves  D, Lehmkuhl K Critical appraisal of systematic reviews of intervention in dentistry published between 2019-2020 using the AMSTAR 2 tool. Evid Based Dent.

[19] Schellinger J, Sewell K, Bloss JE, Ebron T, Forbes C (2021). The effect of librarian involvement on the quality of systematic reviews in dental medicine. PloS One.

[20] Aromataris E, Munn Z (2020). JBI Manual for Evidence Synthesis. Joanna Briggs Institute. Joanna Briggs Institute.

[21] Saltaji H, Armijo-Olivo S, Cummings GG, Amin M, Flores-Mir C (2017). Randomized clinical trials in dentistry: Risks of bias, risks of random errors, reporting quality, and methodologic quality over the years 1955-2013. PloS One.

[22] Perry R, Whitmarsh A, Leach V, Davies P (2021). A comparison of two assessment tools used in overviews of systematic reviews: ROBIS versus AMSTAR-2. Syst Rev.

[23] Wagner CS, Whetsell TA, Mukherjee S (2019). International research collaboration: Novelty, conventionality, and atypicality in knowledge recombination. Res Policy.

[24] Ahn R, Woodbridge A, Abraham A, Saba S, Korenstein D, Madden E (2017). Financial ties of principal investigators and randomized controlled trial outcomes: cross sectional study. BMJ (Clinical research ed. ).

[25] Martinson BC, Anderson MS, de Vries R (2005). Scientists behaving badly. Nature.

[26] Zhang Y, Akl EA, Schünemann HJ Using systematic reviews in guideline development: the GRADE approach. Res Synt Methods.

[27] Neill US (2008). Publish or perish, but at what cost?. J Clin Investig.

[28] Else H, Van Noorden R (2021). The fight against fake-paper factories that churn out sham science. Nature.

[29] Bohannon J (2013). Who's afraid of peer review?. Science (New York).

[30] Van Noorden R (2021). Hundreds of gibberish papers still lurk in the scientific literature. Nature.

[31] Fanelli D (2010). Do pressures to publish increase scientists' bias? An empirical support from US States Data. PloS One.

[32] Tiokhin L, Yan M, Morgan T (2021). Competition for priority harms the reliability of science, but reforms can help. Nat Hum Behav.

[33] Sarewitz D (2016). The pressure to publish pushes down quality. Nature.

[34] Manfredini D, Greene CS, J Ahlberg, De Laat A, Lobbezoo F, Klasser GD (2019). Evidence-based dentistry or meta-analysis illness? A commentary on current publishing trends in the field of temporomandibular disorders and bruxism. J Oral Rehabil.

[35] Gates M, Gates A, Duarte G, Cary M, Becker M, Prediger B (2020). Quality and risk of bias appraisals of systematic reviews are inconsistent across reviewers and centers. J Clin Epidemiol.

